# BION-2: Predicting Positions of Non-Specifically Bound Ions on Protein Surface by a Gaussian-Based Treatment of Electrostatics

**DOI:** 10.3390/ijms22010272

**Published:** 2020-12-29

**Authors:** H. B. Mihiri Shashikala, Arghya Chakravorty, Shailesh Kumar Panday, Emil Alexov

**Affiliations:** 1 Department of Physics, Clemson University, Clemson, SC 29634, USA; mhewabo@g.clemson.edu (H.B.M.S.); arghyac@g.clemson.edu (A.C.); spanday@clemson.edu (S.K.P.); 2 Department of Chemistry, University of Michigan, Ann Arbor, MI 48109, USA

**Keywords:** non-specific binding, surface bound ions, electrostatics, Poisson–Boltzmann equation, dielectric constant

## Abstract

Ions play significant roles in biological processes—they may specifically bind to a protein site or bind non-specifically on its surface. Although the role of specifically bound ions ranges from actively providing structural compactness via coordination of charge–charge interactions to numerous enzymatic activities, non-specifically surface-bound ions are also crucial to maintaining a protein’s stability, responding to pH and ion concentration changes, and contributing to other biological processes. However, the experimental determination of the positions of non-specifically bound ions is not trivial, since they may have a low residential time and experience significant thermal fluctuation of their positions. Here, we report a new release of a computational method, the BION-2 method, that predicts the positions of non-specifically surface-bound ions. The BION-2 utilizes the Gaussian-based treatment of ions within the framework of the modified Poisson–Boltzmann equation, which does not require a sharp boundary between the protein and water phase. Thus, the predictions are done by the balance of the energy of interaction between the protein charges and the corresponding ions and the de-solvation penalty of the ions as they approach the protein. The BION-2 is tested against experimentally determined ion’s positions and it is demonstrated that it outperforms the old BION and other available tools.

## 1. Introduction

Ions are an important component of biological systems as they interact with macromolecules and directly participate in a wide range of reactions [1,2,3]. In molecular biology, the ions can be broadly grouped into two categories: mobile ions in the water phase and ions bound to the corresponding macromolecule. The mobile ions in the solvent freely move in response to the electrostatic environment and their major role is to provide screening of electrostatic interactions [4,5]. On the other hand, the ions bound to macromolecules are involved in specific interactions with macromolecular moiety and play roles in catalysis, electron/proton transfer reactions, and structural stability [6,7,8]. In between these two well distinguishable categories are ions that are weakly bound to the macromolecular surface, without being involved in specific chemical interaction, and have a low residential time—the non-specifically surface-bound ions [9]. This work focusses on such a type of ions and reports a new development of an algorithm, the BION-2 algorithm, that predicts the positions of non-specifically surface-bound ions.

The role of non-specifically surface-bound ions in molecular biology is well documented. Thus, Ca^2+^ and Mg^2+^ ions non-specifically bind to backbone phosphate oxygen atoms of nucleic acid [10,11,12], and the binding reduces the electrostatic repulsion between adjacent phosphate groups and, hence, stabilizes pairing and base stacking [13,14]. The non-specifically surface-bound ions were found to be a key regulator for protein-protein binding and pH-dependence of the binding affinity [15], to affect ion-induced filament formation [16], and to alter macromolecular dynamics [17]. Surface-bound ions are essential for reducing the effective net charge of macromolecules and their effect is manifested via the Zeta potential [18,19]. It was demonstrated that accounting for surface-bound ions is crucial for modeling the experimentally measured Zeta potential for various proteins [20]. The list of examples can be extended; however, it is evident that non-specifically surface-bound ions are essential for many biological processes.

Having in mind the importance of non-specifically surface-bound ions in biology, significant efforts were invested to determine or predict their positions. From an experimental point of view, the main obstacle is that such ions have a low residential time and experience large thermal fluctuations. Furthermore, X-ray-based experimental techniques require crystals to be grown, and some of the ions could be simply artifacts of crystal packing and high-salt concentration typically required for growing crystals. On the other side of the spectra are computational models to predict positions of non-specifically surface-bound ions. To the best of our knowledge, the BION [21,22] is the only publicly available resource for predicting such a type of ions (excluding recent work [12] which, however, does not provide web service), while many other predictors deal with specifically bound ions [23,24].

In this work, we report a new development of BION [21,22], the BION-2, which is a method and a web server to predict non-specifically surface-bound ions. The development takes advantage of a Gaussian-based smooth dielectric function in DelPhi [25,26,27,28,29]. This allows the energy function that evaluates the possibility that a given site holds an ion to be made of two important components: (a) the electrostatic energy of interaction between the candidate ion and the charges of the macromolecules and (b) the de-solvation penalty the ion should pay by approaching the macromolecular surface.

## 2. Results and Discussion

The results section is organized as follows. First, we provide two examples of a predicted ion’s positions along with experimentally determined surface-bound ions. Second, we report the results on benchmarking BION-2 to predict surface-bound ions against experimentally determined ions’ positions. Lastly, we compare BION-2 predictions with VMD [30] and FoldX [31] predictions.

### 2.1. The Visual Example Section Outlines Two Cases

**Figure 1:**
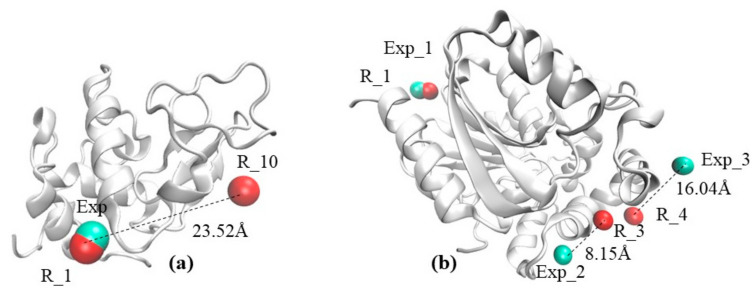
Protein structures in ribbon presentation along with experimentally determined and predicted ions (shown as balls) with its Euclidean distance (dotted line). (**a**) Protein PDB ID 1C10 in white ribbon presentation, with experimentally determined Cl^−^ (cyan ball) and predicted Cl^−^ R_1 and R_10 (red ball); (**b**) protein PDB ID 1IZ7 shown with white ribbon with experimentally determined Ca^2+^ ions (cyan balls) and predicted R_1, R_3, and R_4 (red balls). R_1, R_2, R_4, and R_10 are the predicted ions’ positions using BION-2 and the number indicates the prediction rank. Exp is the label for experimentally determined ions and the number indicates how many ions’ positions were experimentally determined (this is not ranking).

The visual example, (a) a case of a protein with only one experimentally determined ion; and (b) a case of a protein with three experimentally determined ions. The first example illustrates a non-ambiguous case of a protein (listed as 1C10 in PDB) which has only one Cl^−^ ion bound (Figure 1a). The predicted Cl^−^ position with rank 1 (the most confident prediction) closely matches the experimental one, while the less confident prediction with rank 10 is far away from the experimental one (Figure 1a). The second example illustrates a case of a protein (listed as 1IZ7 in PDB) that has three experimentally determined Ca^2+^ ions. The rank 1(R_1) predicted position closely matches one of the experimental ion positions, while the other two experimental positions are matched by predictions with rank 3 (R_3) and 4 (R_4). This case illustrates the details of the benchmarking protocol that will be presented in the next section of the paper—namely, the experimental position labeled as Exp_1 in Figure 1b will be reported to be very successful, while the other two positions Exp_2 and Exp_3 will be reported as rank 3(R_3) and rank 4 (R_4), respectively.

### 2.2. Benchmarking of BION-2 Performance

Here, we use two quantities to assess the performance of BION-2, the distance between the experimentally determined ion position and the rank 1-predicted ion position and the shortest distance between the experimentally determined ion position and any of top ten predicted positions independent of their rank (D_min_). The first quantity provides a measure of the ability of BION-2 to correctly predict the ion position (however, see the above example with multiple ion positions around the same protein), while the second quantity benchmarks the ability of the BION-2 algorithm to generate appropriate positions within the top 10 ranked positions. Note that, in the case of multiple experimentally determined ion positions, we choose the position closest to the predicted one in the benchmarking and thus provide better assessment of the accuracy of the predictions in case of multiple ion positions around the same protein.

The optimal performance is expected to result in the smallest difference between the experimentally determined ion position and the predicted one with rank 1, as well as the smallest D_min_ (in case of multiple ion positions in the same protein). To test the sensitivity of predictions with respect to the grid resolution, the value of internal reference dielectric constant and the ion concentration were systematically varied. The best performance was achieved at an internal reference dielectric constant of 2 and a salt concentration of 0.5M. A tradeoff between the resolution and the speed of calculations was reached at a grid resolution of 2 grids/Å. The rest of the results are reported for this set of parameters which were made the default for the BION-2 algorithm and web server.

**Figure 2:**
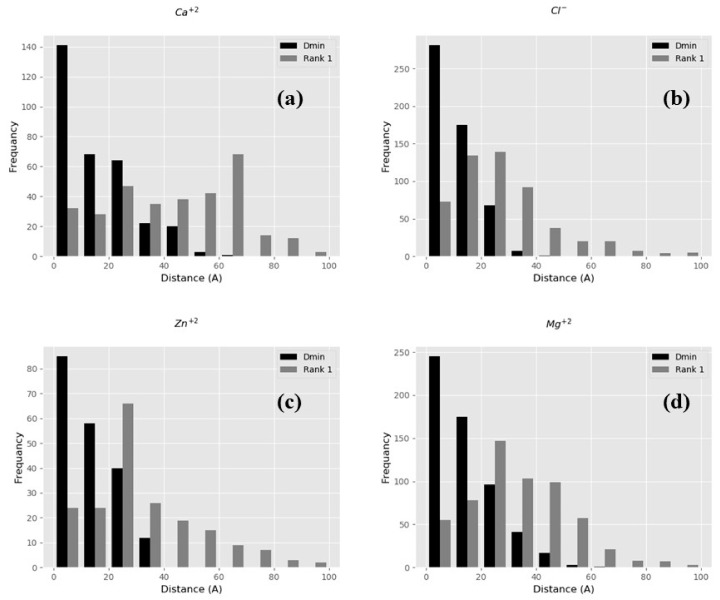
Benchmarking results for predicted ion’s positions as compared with experimental ones. Distance distribution with rank 1 (gray bars) and the closest distance (D_min_) between the original ion’s position and predicted position (black bars). The distribution of the number of cases (frequency along y-axis) vs. Distance (x-axis) for ion types Ca^2+^, Cl^1−^, Zn^2+^, and Mg^2+^ are shown in panels (**a**–**d**), respectively.

The experimental dataset provides cases for four types of ions, and benchmarking results are shown in Figure 2. It can be seen that the distribution of D_min_ is much more impressive than the distribution of the rank 1 distance. Indeed, many of the experimental cases are proteins with multiple ion positions. Despite that, one can see that about 10% of Ca^2+^, Cl^−^, and Zn^2+^ ions’ positions are predicted accurately by the rank 1 prediction. If one provides a tolerance of 20 Å, then about 80% of Cl^−^, Mg^2+^, and Zn^2+^ ions’ positions are predicted accurately as well.

Since the experimental dataset is identical to the dataset used in the previous BION version [22], we compared the performance of the new BION-2 with the old version of BION, which uses traditional the Poisson–Boltzmann equation (PBE) and the standard treatment of molecular surface. Results are shown in Figures S1 and S2. One can see that the new BION-2 outperforms the old BION version in both ranking and predicting positions with a smaller D_min_. This is especially notable for the ranking of Ca^2+^ and Zn^2+^ ions, where BION-2 is much more accurate than the old BION version.

#### 2.2.1. BION-2 vs. VMD

**Figure 3:**
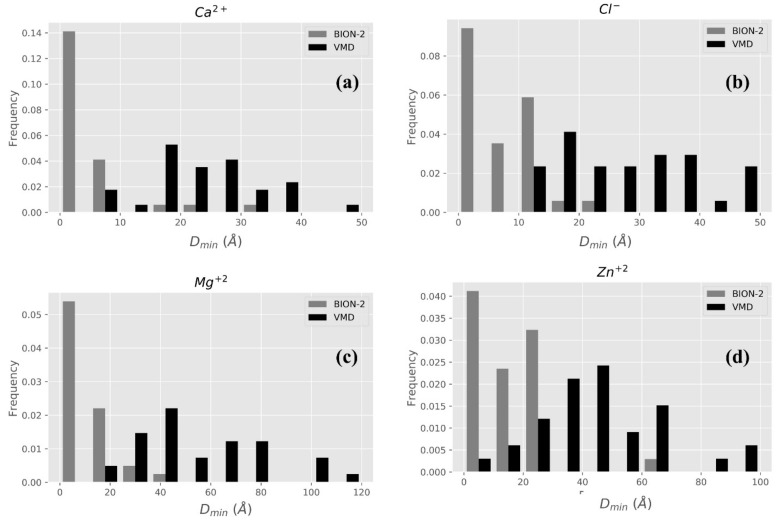
Distribution of the best VMD predictions (black bars) and best BION-2 predictions (gray bars). The distribution of normalized frequency (*y*-axis) vs. D_min_ (*x*-axis) for ion types Ca^2+^, Cl^−^, Mg^2+^, and Zn^2+^ are shown in panels (**a**–**d**), respectively.

Albeit the “ionize” module of VMD is designed to place ions in solution, at a distance no less than 6 Å away from the protein surface, and that the goal is to neutralize the net charge of the protein, it is still tempting to compare VMD with BION-2 predictions (the VMD requirement of placing the ions at more than 6 Å away from protein surface is tolerable since many of BION-2 predictions are within the same range—Figure 2). It should be mentioned that VMD does not rank ion positions, thus if VMD needs N ions to be placed to neutralize the system, they are placed without ranking. Therefore, in favor of VMD, we select among these N ions the ion closest to the experimentally determined position. In the case of BION-2, we apply the same protocol and select the best results within rank 1 to rank N (the same number of ions placed by VMD for this particular protein). Results are shown in Figure 3. It can be seen that BION-2 predictions are much closer to experimentally determined ions’ positions than those of VMD.

#### 2.2.2. BION-2 vs. FoldX

**Figure 4:**
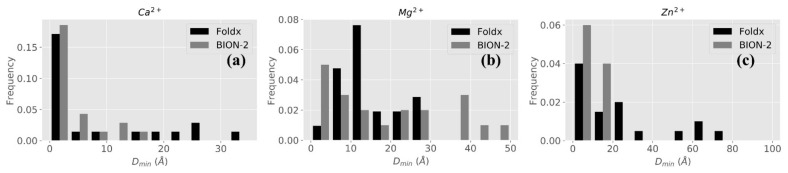
Distribution of the best FoldX predictions (black bars) and best BION-2 predictions (gray bars) with normalized frequency on y-axis and D_min_ on x-axis for ion types Ca^2+^ (**a**), Mg^2+^ (**b**), and Zn^2+^ (**c**) are shown.

Figure 4 shows the comparison between BION-2 and FoldX. Benchmarking results for Cl^−^ are not compared because FoldX is designed to predict positions of metal ions only. It can be seen that BION-2 predictions are significantly better than those of FoldX, since the number of ions predicted by BION-2 which are within very short distances from experimental positions is larger. This is particularly clear for Mg^2+^ and Zn^2+^ ions (Figure 4).

#### 2.2.3. Computational Efficiency

The BION-2 algorithm works in two steps (i) DelPhi is run to generate the potential map, then the (ii) points on the potential map are ranked using heap-sort. DelPhi uses an iterative Gauss–Seidel algorithm with a time complexity of O(n^3^), where n is proportional to the largest dimension (in Å) of the input protein and is the side length of the computing box. Later, the heap-sort technique is used to sort and rank each of the n^3^ grid point rendering a time complexity of O(n^3^ log n). To provide insight, additionally, we compare the computational time by BION-2 and FoldX considering 10 cases. The average computational time is reported by considering the Zn^2+^ and Mg^2+^ ions predictions in three runs for each case (Table 1). It can be seen that the BION-2 computational time is significantly lower than that of FoldX. 

#### 2.2.4. BION-2 Webserver

The method is implemented into a webserver that is freely available to the community. The users must provide a structural file in PQR format and select the ion type and number of ions to be predicted. The BION-2 returns the position (*x*,*y*,*z* coordinates) of the predicted ions along with visualization and other relevant information. 

## 3. Materials and Methods

### 3.1. Database of Protein Structures

To benchmark BION-2 predictions, we used a previously compiled set of X-ray structures with surface-bound ions [9,22] (http://compbio.clemson.edu/downloads DatabaseFixProOrig.tar.gz). An attempt was made to include NMR structures as well, but we were unable to find surface-exposed ions. The X-ray dataset comprises 446 proteins in total, including 47, 29, 153, and 224 proteins and 51, 35, 161, and 267 ions for Ca^2+^, Zn^2+^, Cl^−^, and Mg^2+^, respectively.

### 3.2. Ions’ Treatment in the Framework of Gaussian-Based Smooth Dielectric Function

In the Gaussian-based smooth dielectric model, the solute and solvent are treated on the same footage via a smooth Gaussian-based dielectric function. It ensures that a smooth transition of the dielectric properties occurs from the macromolecular interior to the water phase. The idea is to represent each atom as an atom-centered Gaussian density function (Equation (1)) as opposed to a hard sphere [26,28,29,32].

(1) $$g_{i}(\vec{r})=\;\exp[\frac{-{\left|\vec{r}-{\vec{r}}_{i}\right|}^{2}}{\sigma^{2}R_{i}{}^{2}}]$$

where ${\vec{r}}_{i}$ is the center of the *i*th atom, *R_i_* is the van der Waals radius of the *i*th atom and σ is the relative variance. Then, the cumulative density function ($g(\vec{r})$) for multiple atoms is given by:

(2) $$g(\vec{r})=1-\prod_{i=1}^{N_{m}}\left[1-g_{i}(\vec{r})\right]$$

where *N_m_* stands for the total number of atoms. 

In the end, the smooth dielectric function throughout the space is defined as:

(3) $$\epsilon (\vec{r})=g(\vec{r})\epsilon_{in}+(1-g(\vec{r}))\epsilon_{w}$$

where, $\epsilon_{in}$ and $\epsilon_{w}$ are internal and external reference dielectric constants in the macromolecule and water, respectively.

Since there is no sharp boundary between the solvent and solution in the Gaussian-based smooth dielectric function model, the treatment of mobile ions in the water phase requires the modification of the Poisson–Boltzmann equation (PBE), so as not to allow ions to penetrate into the solute interior. Recently, we introduced a modified PBE that penalizes ions to be present in regions close to protein atoms by adding a de-solvation penalty term within the Boltzmann factor [27]:

(4) $$\nabla \cdot \left[\varepsilon \left(r\right)\nabla \varphi \left(r\right)\right]=-4\pi \left(\rho_{solute}\left(r\right)+\overset{N}{\underset{i=1}{\sum }}q_{i}c^{bulk}exp\left(\frac{-q_{i}\varphi \left(r\right)-\Delta G_{solv}}{RT}\right)\right)$$

where $\varepsilon \left(r\right)$, $\varphi \left(r\right)$, and $\rho_{solute}\left(r\right)$ are the dielectric constant, electrostatic potential, and charge density of solute in space, respectively, −$q_{i}$ is the ionic charge, $c^{bulk}$ is the ion concentration in bulk solvent, $\Delta G_{solv}$ is the solvation penalty term for ions, *R* is the ideal gas constant, and *T* is the temperature. The de-solvation penalty, Δ*G_solv_*, is calculated using the following formula:

(5) $$\Delta G_{solv}=\frac{N_{A}z^{2}e^{2}}{8\pi \varepsilon_{0}r_{0}}\left(\frac{1}{\varepsilon_{r}}-\frac{1}{\varepsilon_{w}}\right)$$

where $N_{A}$ is the Avogadro constant, *z* is the valence of the ion, *e* is the elemental charge, $\varepsilon_{0}$ is the permittivity of vacuum, $r_{0}$ is the effective radius of the ion, $\varepsilon_{r}$ is the dielectric constant at a given location, and $\varepsilon_{w}$ is the dielectric constant of bulk water. For computational efficiency, the effective ion radius is approximated using 2.0 Å for both cations and anions. 

### 3.3. Electrostatic Potential Map Calculations

Electrostatic potential 3D distribution (electrostatic potential map) was obtained with DelPhi applying the following parameters: scale = 2 grid/Å; percent of protein filling of the cube = 70%; Gaussian-based smooth dielectric function; a reference dielectric constant of 2 for the protein; and 80 for the solvent; the ionic strength was varied from 0.1 to 0.5 M. Ions and all other hetero atoms were deleted form the corresponding PDB files. 

### 3.4. Algorithm for Predicting Ion’s Position

The predicting algorithm utilizes a DelPhi-calculated electrostatic potential map and analyzes all grid points outside the van der Waals (vdW) surface of the protein. The decision of placing an ion at a given position is based on the energy formula, provided below, that combines the strength of the electrostatic interactions between the ion and protein and adds the de-solvation penalty for the ion. This term reduces the solvation energy when the ion is close to protein atoms where $\varepsilon_{r}$ is low.

(6) $$G\left(s\right)=q_{ion}\Phi \left(s\right)+\frac{N_{A}z^{2}e^{2}}{8\pi \varepsilon_{0}r_{0}}\left(\frac{1}{\varepsilon_{r}}-\frac{1}{\varepsilon_{w}}\right)\;$$

One can note that this is the argument of the Boltzmann factor in the corresponding modified PBE (Equation (4)). Thus, the first term in Equation (6) is the energy of interaction between protein charges and the ion determined by the product of the electrostatic potential (Φ(*s*)) calculated at each grid point and the charge of the ion (*q_ion_*). The second term is the de-solvation penalty, Equation (5), where $\varepsilon_{r}$ is the averaged dielectric value at the corresponding grid point, averaged over the six neighboring midpoints. This formalism, i.e., Equation (6), is along the lines of the Gaussian-based approach in DelPhi that does not consider a sharp border between solute and solvent, and instead assigns a smooth de-solvation penalty function to prevent ions from going inside the solute. 

The Equation (6) is used to assign value *G*(*s*) for each grid point outside the vdW surface of the solute (note that the grid points near the solute will have the highest *G*(*s*), since the electrostatic potential quickly decays away from the protein charges). Then, a heap-sort technique is used to rank each grid point on the basis of the corresponding *G*(*s*), resulting in a priority queue. The most prior, and therefore the most highly ranked site, is the one with the lowest value of $G\left(s\right)$ (note that a negative value makes the energy favorable). To reduce memory usage, only sites with a negative value of $G\left(s\right)$ are stored and the rest is discarded.

From the priority queue, sites are “popped” in the order they are stored to check for plausible vdW clashes. Thus, each “popped” site’s prospect of steric clash with the protein’s vdW surface is measured by comparing its distance from the nearby protein atoms ($r\left(S,A\right)$) and the sum of their vdW radii ($R_{ion}$ and $R_{atom}$), i.e., a site is discarded if $r\left(S,A\right)<R_{ion}+R_{atom}$. If successful, the site is then checked for its proximity to all the other predicted sites by ensuring that the distance between the two is greater than 6Å ($≳2R_{ion}$). If a site successfully passes these two tests, it is listed as a prospective site and assigned a new rank. As mentioned above, the lower the rank, the better suited a site is to hold an ion in question around that protein. 

The number of prospective sites output by the refined program is limited by a maximum, which a user can provide. For each output site, their coordinates and ranks are printed. To help with further analyses, the outputs also report the site’s dielectric, de-solvation energy therein and a list of the neighboring protein atoms. 

### 3.5. Using VMD to Place Ions

We use the VMD-ionize module to compare the VMD’s and BION’s predictions for ions’ positions (for given type of ions). VMD-ionize is a program for placing ions near a biological molecule in preparation for molecular dynamics simulations to make sure the net charge of the system is zero. In this case, the placement is performed by calculating the coulombic potential due to the molecule in the nearby volume and placing ions at points of minimal energy. After each ion is placed, the potential is updated, so that subsequent ions will be placed in response to this. It should be mentioned that VMD places ions at distances no less than 6 Å away from the protein surface, so it is not intended to predict the positions of surface-bound ions. However, we use VMD-placed ions to compare with BION-2-placed ions to get an idea of how important it is to calculate the electrostatic potential via PBE instead of using Coulomb’s law in a homogeneous media, and also emphasize the importance of the desolvation penalty term.

### 3.6. Using FoldX to Predict Ions’ Positions

FoldX [31] predicts only metal ions which are Ca, Mg, Mn, Na, Zn, Fe, Cu, Co, and K and produces an output if a high affinity metal binding site is predicted. We choose −3 kcal/mole (default value, lowAffinityMetal = −3) as the threshold energy while predicting ions sites.

## 4. Conclusions

A new development of the BION algorithm, the BION-2, was reported and shown to outperform the old one in placing non-specifically surface-bound ions. While placement of ions in the solution is a standard procedure prior to an Molecular Dynamics (MD) simulation, and there are many tools for doing that, we demonstrated that they are not efficient in predicting surface-bound ions. Thus, if one is concerned with predicting surface-bound ions, BION-2 should be the primary choice. The method is available as a web server as well at http://compbio.clemson.edu/BION-2/. 

## Figures and Tables

**Table 1:** The average computational time for 10 cases. The computational time is provided in seconds.

PDB	FoldX(s)	BION-2 (s)	No. of Residues	Ion Type
1L9A	5.0	3.0	87	Mg^+2^
1QGW	33.0	9.0	176	Mg^+2^
1E2D	23.0	6.0	215	Mg^+2^
1NG1	19.0	14.0	294	Mg^+2^
1LR0	9.0	6.7	125	Zn^+2^
2CEI	27.7	9.3	183	Zn^+2^
2AS9	72.7	12.0	210	Zn^+2^
1ET5	54.0	16.0	341	Zn^+2^
1TY3	22.0	14.0	357	Zn^+2^
3HK5	205.0	65.0	427	Zn^+2^

## Data Availability

Data is available at http://compbio.clemson.edu.

## Supplementary Materials

The following are available online at https://www.mdpi.com/1422-0067/22/1/272/s1, Figure S1: Surface bound ions D_min_ comparison with Old and New BION-2 for X-ray structures, Figure S2: Surface bound ions Rank1 comparison with Old and New BION-2 for X-ray structures; and list of X-ray structures.

## Abbreviations

BION	bound ion prediction method
Zeta	Zeta potential
PDB	Protein data bank
DelPhi	Poisson–Boltzmann solver
VMD	visual molecular dynamics
PBE	Poisson–Boltzmann equation
vdW	van der Waals
PQR	position, charge, radius format
NMR	nucleic magnetic resonance
