# Supplementary material for: BION-2: Predicting Positions of Non-Specifically Bound Ions on Protein Surface by a Gaussian-Based Treatment of Electrostatics
Source: Int J Mol Sci. 2020 Dec 29;22(1):272. doi: 10.3390/ijms22010272 (PMC7794834; doi:10.3390/ijms22010272)
Supplement: Supplementary file 1 [file ijms-22-00272-s001.pdf]

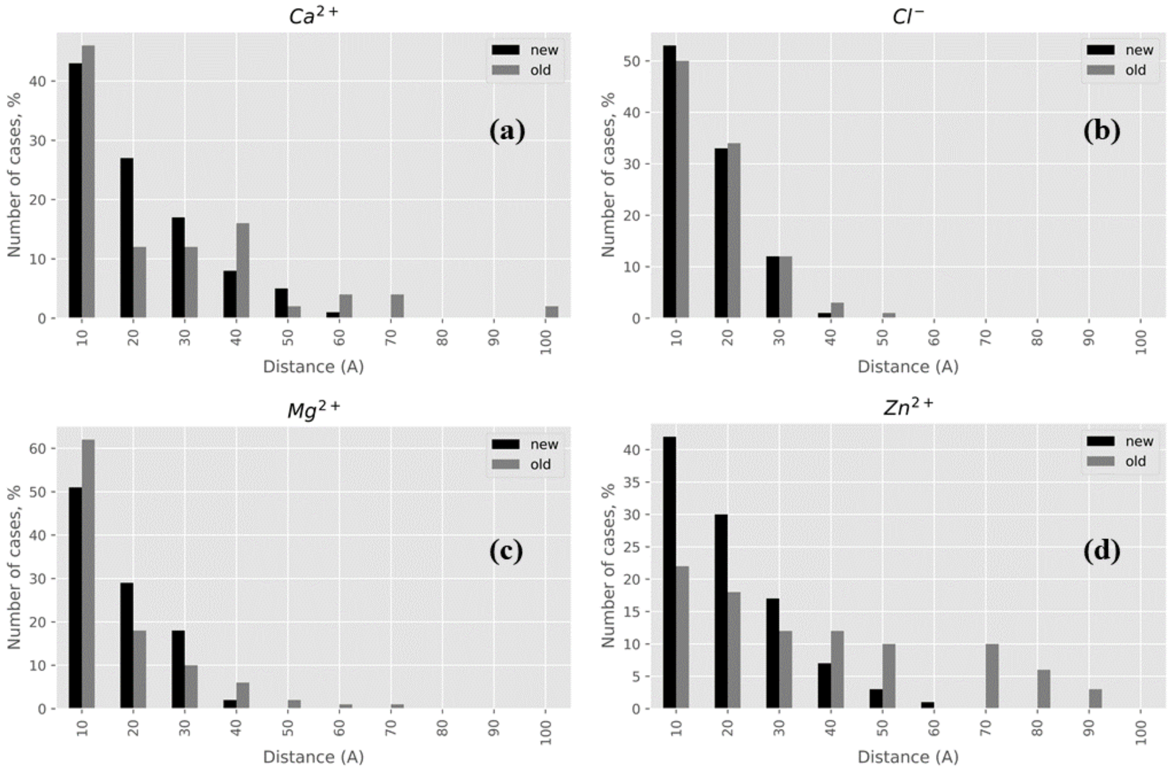

Figure S1. Surface bound ions D_{min} comparison with Old and New BION-2 for X-Ray structures.

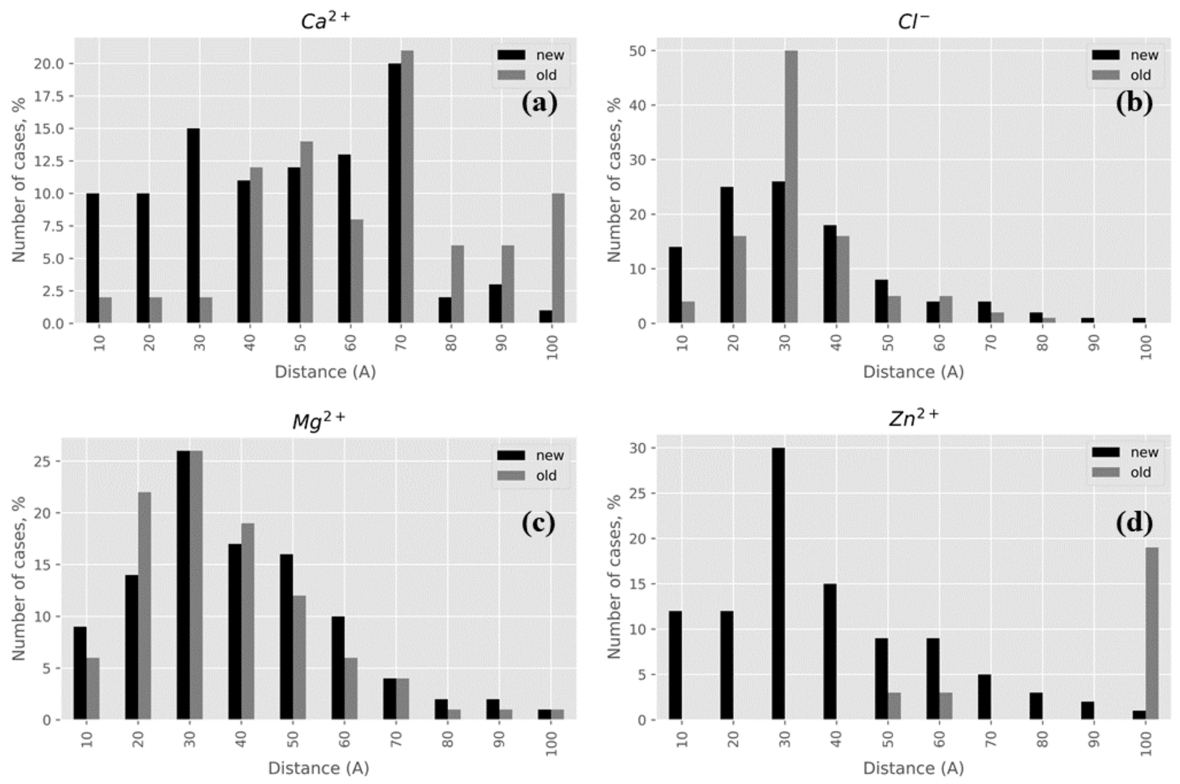

Figure S2. Surface bound ions Rank1 comparison with Old and New BION-2 for X-Ray structures.

List of X-ray structures:

Ca^{2+}

1BVI 1DDS 1EZJ 1G4H 1G5F 1GUN 1I0X 1IZ7 1IZ8 1KWT 1KWU 1KWW 1KWX 1KWY 1KYT 1M5K 1M5O 1M5V 1RX1 1T64 1U94 1V3W 1V6C 1XRX 1YLI 2A11 2BFN 2FXU 2GXS 2I52 2KMB 2OZB 2PYH 3B3D 3B7E 3BEQ 3BWX 3DE8 3E3R 3HDB 3HOH 3IKV 3KLT 3LWA 3LYB 4KMB 5HOH

Cl^{-}

160L 191L 193L 194L 1AT5 1C10 1C5Q 1C69 1C6A 1C6B 1CE5 1DPW 1DPX 1DYA 1DYB 1DYC 1DYD 1DYE 1DYF 1DYG 1G71 1GJV 1H3M 1IEP 1IHN 1JFX 1KLZ 1L36 1L4Y 1L57 1L69 1L70 1L71 1L72 1L73 1L74 1L75 1L96 1LZ8 1MG1 1MOG 1O3B 1ORU 1P0P 1SZ6 1T3P 1TJB 1U16 1V16 1V9C 1VAU 1W15 1XAG 1Y4H 1YIL 1YKX 1YKY 1YKZ 1YLF 1Z55 1ZKX 1ZL5 1ZUR 1ZYT 223L 226L 2A5A 2A7F 2BI4 2C4H 2DT5 2FBM 2FJ1 2FK5 2G4P 2G4V 2G4Z 2GHW 2GNN 2HX1 2I4G 2NTE 2P67 2Q02 2Q0M 2QX8 2RG9 2RKI 2UXN 2V79 2V84 2VRE 2VX9 2W4M 2WHY 2WID 2WIG 2WML 2WQ9 2XKC 2XNN 2Z0R 3A34 3A8Z 3A90 3AEI 3AGG 3AGH 3B72 3BFB 3BFT 3C7W 3C7Z 3C80 3C81 3C83 3C8Q 3C8R 3CDR 3CDT 3CDV 3CKP 3D0K 3DA2 3DJI 3DKK 3EMS 3FOM 3FSG 3FUA 3G3X 3GG3 3H6C 3HKY 3I2G 3I3N 3I8W 3K2J 3KG1 3KVW

3L27 3MS9 3NCP 3OJP 3PKA 3PKB 3Q0P 3RNX 3SP3 4LZM 5DFR 6LZM 7LZM

Mg^{2+}

1AAX 1AIH 1BVW 1DOZ 1E0C 1E2D 1E2E 1E2F 1E2G 1E2Q 1E9A 1E9B 1E9C 1E9D 1E9E 1E9F 1EEN 1EK6 1FEU 1H3J 1I57 1IIR 1J1Y 1J3W 1K03 1K5P 1K5P 1K63 1K6E 1L2E 1L9A 1LJ0 1LO6 1LYK 1MZY 1N70 1NA0 1NA3 1NG1 1NMX 1NMZ 1NN0 1NN1 1NN3 1NN5 1NWL 1NYK 1OET 1OEV 1OYJ 1PTY 1PXH 1Q6N 1Q6S 1Q6T 1QGW 1QV9 1QV9 1R2R 1R3C 1SS4 1T49 1U8Y 1UIK 1UQZ 1W5M 1XCM 1XD3 1XJ0 1YJ2 1YM0 1YNS 1Z08 1ZKE 1ZS9 1ZUK 1ZV2 1ZV2 2A3T 2AWM 2BH3 2BHA 2BHB 2BHC 2BHD 2BHX 2BI1 2BI2 2BI3 2BI5 2BI9 2BIA 2BN7 2BWT 2BWU 2BWW 2BWY 2C4E 2CC9 2CL0 2CM8 2CNF 2CNG 2CNI 2CZ6 2D0Q 2DE3 2DSL 2DY0 2ERB 2F6F 2F6M 2F6T 2F6V 2F6W 2F6Y 2F6Z 2F70 2F71 2FQ1 2FWQ 2G0W 2G5Z 2GPC 2H03 2HB4 2HBV 2HIY 2HK6 2HSJ 2HYR 2I5U 2IBJ 2IBP 2IK9 2IK9 2NUG 2ODA 2OJ6 2OY9 2PRY 2Q2N 2Q2N 2QIN 2QMQ 2R2H 2RDN 2RDQ 2RDS 2RE9 2RFG 2V1P 2VEV 2VEW 2VEX 2VK6 2VWX 2VWY 2VWZ 2VX0 2VX1 2VXT 2WCH 2WCJ 2WCK 2WCL 2WCM 2WQS 2XCL 2XD4 2XGZ 2ZFG 2ZPB 2ZPE 2ZPF 2ZPG 2ZPH 2ZPI 3A64 3ABX 3AJO 3BE6 3BZ7 3BZ7 3C2T 3C2T 3C3I 3C3I 3CA9 3CON 3CU3 3CYZ 3CZ0 3DNP 3DUU 3DV6 3EDV 3EF8 3EZX 3FVB 3FWZ 3G0S 3G0S 3G1T 3GOQ 3GW1 3H7F 3HZV 3IMH 3IWT 3K1E 3K1U 3K6Q 3LBI 3LBN 3LIE 3LM8 3M07 3MEL 3NER 3NYQ 3O69 3P5P 3QGP 3QVQ

Zn^{2+}

1BYF 1EBO 1ET5 1GHY 1LR0 1S03 1TLG 1YT3 2AS9 2BOQ 2CEI 2CHI 2D0W 2FAD 2FS5 2IU2 2JJL 2O6E 2VH5 3BYR 3FGG 3H6T 3HK5 3HK8 3IET 3M6R 3M9G 3PSQ
